# State Evaluation Method of Robot Lubricating Oil Based on Support Vector Regression

**DOI:** 10.1155/2021/9441649

**Published:** 2021-09-13

**Authors:** Dongdong Guo, Xiangqun Chen, Haitao Ma, Zimei Sun, Zongrui Jiang

**Affiliations:** ^1^Technical Service Site, Beijing Benz Automotive Co. Ltd., Beijing 100176, China; ^2^School of Software & Microelectronics, Peking University, Beijing 100871, China

## Abstract

Recently, the development of the Industrial Internet of Things (IIoT) has led enterprises to re-examine the research of the equipment-state-prediction models and intelligent manufacturing applications. Take industrial robots as typical example. Under the effect of scale, robot maintenance decision seriously affects the cost of spare parts and labor deployment. In this paper, an evaluation method is proposed to predict the state of robot lubricating oil based on support vector regression (SVR). It would be the proper model to avoid the structural risks and minimize the effect of small sample volume. IIoT technology is used to collect and store the valuable robot running data. The key features of the running state of the robot are extracted, and the machine learning model is applied according to the measured element contents of the lubricating oil. As a result, the cost of spare parts consumption can be saved for more than two million CNY per year.

## 1. Introduction

In the age of Industry 4.0, artificial intelligence is gradually changing the manufacturing process. Thanks to the development of Industrial Internet of Things (IIoT), monitoring physical processes has become an applicable implementation, which enables us to make extensive data-driven decisions through machine learning methods. Real-time communication between sensors, machines, and people can reduce the equipment breakdown time, detect production flaws, and optimize supply chains. All these steps can be even improved by learning from historical experience. Meanwhile, industrial robots play an increasingly important role in the wide-range fields, as they are used in almost all aspects of manufacturing to improve productivity and reduce costs. However, when the robot fails on the production line, it is very likely to have a negative impact on the entire production process. In an even worse case, it may lead to a line halt until the problem is solved, which will result in huge economic losses due to the unpredictable breakdown time.

Therefore, the maintenance of robot is indispensable for good reliability. The traditional way of maintenance is so-called “run-to-failure” which only performs after the occurrence of malfunction [1]. The other method “preventive maintenance” is mostly based on the periodic replacement of spare parts, which is easy to cause excessive or insufficient maintenance. Consequently, the traditional maintenance consumes additional spare parts resources and takes up large labor cost that is no longer suitable for the development of Industry 4.0. In order to achieve the predictive maintenance, the utilization of extra sensors is necessary to collect real-time data. However, it is not practicable in many circumstances due to the added cost, system complexity, and the requirement of additional space within the robotic system [2]. In this aspect, the predictive and preventive maintenance based on machine learning algorithm has attracted a great deal of attention in recent years. The internal signal of robot (e.g., motor torque) plays as an important role in maintenance which can reflect the robot working condition. In the existing algorithm, the features of internal signals are learnt and extracted from the robots with healthy states. They are used to establish a model that can represent the normal robot status. Thereafter, the model is tested on the running process to detect any variations in the mechanical condition of the robot, which may indicate the predictive problems [3]. Furthermore, any complicated system with unknown disturbances can be tracked by an adaptive scheme to analyze the primary causes [4].

Due to the increment of intelligent equipment complexity in the manufacturing industry, the corresponding software promotes the close integration of information technology (IT) and operational technology (OT) and thus creates opportunities to gain new insights and observation perspectives from the original knowledge. IIoT would force the scientists to focus on the device connectivity, data analytics, process optimization, and intelligent operations. The manufacturer may desire to set up the integrated management, control platform, and big data center in order to achieve the convergence of IT and OT. The data are used to develop production or business models, simulation analysis, and interactive display to reach the data-driven value. By using the horizontal integration of process and production as well as the vertical connection of workshop equipment and adopting the technologies of digitalization and virtualization, the factory operation monitoring and equipment health management are accomplished. The achievement of process/production line optimization based on the quality data is highly desired, and thus the efficient and rapid production can be realized. Furthermore, the production and operation costs can be significantly reduced.

Thanks to the development of IIoT, machine learning algorithms are available to utilize as the predictive model, including survival analysis, extremely randomized trees, *k*-nearest neighbors, and convolutional neural network (CNN) [5–8]. Take the research from Susto et al. as typical example [1]. In order to compare the performance of preventive maintenance and predictive maintenance, an issue from semiconductor industry was chosen as the benchmark. Generally, the ion implantation was considered as the bottleneck in the semiconductor production line due to the replacement of tungsten filaments, which had to be frequently changed and each time it may stop the production for approximately three hours. The working lifetime of filaments was probably influenced by several factors that can be utilized as input for the classifier model. Herein, the support vector machine (SVM) and k-nearest neighbors (k-NN) were applied as the multiple classifiers to evaluate the health status of samples. After the optimization, the build-up predictive maintenance system was demonstrated as an efficient method to improve the maintenance decision and minimize the operating costs.

In addition to machine learning algorithms, different ways were introduced to predict a robot failure in previous research [9–15], which mainly focused on robot motors. However, the status of robot gearbox lubricating oil is equally important as it affects the robot motor operation, gearbox wear, and maintenance cost, which may lead to spending millions of CNY for the entire plant replacement. Lubricating oil is the critical component of drive system that normally determines the working lifetime of entire equipment. Most of the bearings and gears are operating based on the thin oil film as it can significantly reduce the friction and avoid wear [16]. It is proved that the robot and motor properties are highly related with the oil condition, and thus a method is possible to be developed for oil evaluation through exterior robot [17].

Nevertheless, the theory of robot status that represents the operating function of lubricating oil is still a mystery. Meanwhile, it is hard to regularly check the oil condition as it is not the consumable item and takes excess labor cost. Therefore, an optimized model is highly desired to evaluate the health condition of oil through real-time data analysis instead of physical measurement. Regarding the current industrial circumstance, the key features of the robots (e.g., torque and temperature) may be considered as the independent variables that can be easily collected by IIoT data acquisition technology. Their variation possibly resulted from the gear box oil based on the complicated relationship. Since the brand-new lubricating oil does not contain any iron, the additional iron element content would be considered as the dependent variable. It should be mainly generated by the wear and tear of robot that can represent the performance of lubricating oil. The experiential and analytical knowledge are necessary to apply the predictive maintenance.

Herein, the present study contributes to establishing the evaluation method of lubricating oil condition based on the measurable robot properties. A machine learning model would be applied which can assess the lubricating performance and forecast the maintenance strategies without extra oil consumption or labor cost. Therefore, the study narrows down the maintenance scope that focuses on the robots in need. This project would probably save millions of money in terms of spare parts replacement.

This paper is organized as follows. The data acquisition, machine learning model, and research workflow are introduced in Section 2. The model performances and analysis are given in Section 3. The summarized conclusion is provided in Section 4.

## 2. Materials and Methods

### 2.1. Data Acquisition Framework

In order to achieve the practical model, all the data are acquired from on-site production. Message Queuing Telemetry Transport (MQTT), which was proposed by IBM in 1999, is a messaging protocol and uses publish-subscribe method for communication. The original purpose was designed for unreliable communication between limited memory devices and networks with low network bandwidth. It is very suitable for Internet of Things communication.

**Figure 1:**
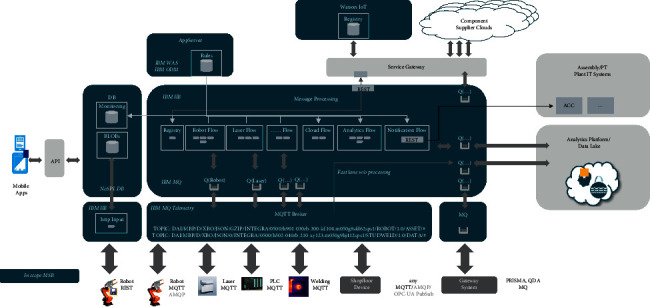
BBAC IIoT platform framework.

Based on MQTT protocol, a non-SQL database and a Hadoop ecosystem were employed by Beijing Benz Automotive Co. Ltd. (BBAC) as real-time data display and Data Lake, respectively, to construct industrial Internet of Things system, as shown in Figure 1.

Based on the MQTT protocol, the developed client is installed in the robot operating system, and the robot data can be sent by subscription. Considering the processing ability, network load, and data storage ability of the robot system, BBAC configures the data transmission period of the robot at a frequency of 0.5 Hz (one point for every two seconds). The data include robot name, program name, running time, torque of each axis, temperature of each axis, current trajectory coordinate, and other information.

### 2.2. The Support Vector Regression (SVR) Model

Vapnik (1995) introduced the support vector machine (SVM) theory [18]. There are two main categories for SVM: support vector classification (SVC) and support vector regression (SVR). SVR is the most commonly used application form of support vector machine, which was put forward by Vapnik in 1997. The SVR model is defined by the hyperplane, regression bound, and support vectors. The main purpose is to evaluate the functional relationship between input and output random variables under the presumption that the joint distribution P of input and output variables is entirely unknown. Especially, it defines a prediction line of the target feature to represent the hyperplane and use a tolerance defined by the support vector to represent the regression bound. Thus, the algorithm of the SVR model optimizes the model parameters to minimize the prediction error and maximize the tolerance as well. Moreover, it is able to hybridize with empirical mode for complex system forecasting [19].

**Figure 2:**
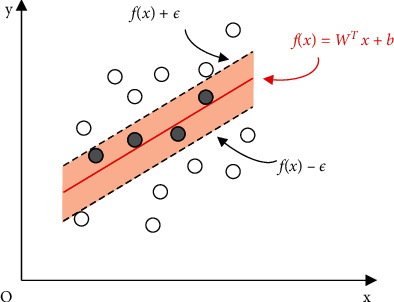
Regression estimation function *f*(*x*).

Regression estimation can be transformed into a problem of inferring function *f* (*x*). Given a training set  *D* = {(*x*_1,_, *y*_1_), (*x*_2_, *y*_2_),…, (*x*_*n*_, *y*_*n*_)},  where *y*_*i*_ ∈ *R*, the goal is to make*f* (*x*) and *y* as close as possible. *w* and *b* are the model parameters to be identified. Assuming we can tolerate the maximum deviation between *f*(*x*) and *y*, the loss is computed only when the absolute value of the difference between *f*(*x*) and *y* is bigger than ɛ (as shown in Figure 2).

Therefore, the SVR problem can be formalized as

(1) $$\begin{array}{c} \underset{w,b}{\min}\frac{1}{2}{\left|\left|w\right|\right|}^{2}+C\overset{m}{\underset{i=1}{\sum }}l_{\varepsilon }\left(f\left(x_{i}\right),y_{i}\right), \end{array}$$

where *C* is the regularization constant and *l*_*ε*_ is the insensitive loss function.

(2) $$\begin{array}{c} l_{\varepsilon }=\left\{\begin{array}{cc} 0,\; & \text{if }\left|z\right|\leq \in ,\\ \left|z\right|-\in , & \text{otherwise.} \end{array}\right. \end{array}$$

Introducing relaxation variables *ξ*_*i*_ and $\hat{\xi_{i}}$, the formula can be rewritten as

(3) $$\begin{array}{c} \underset{w,b,\xi_{i},\hat{\xi_{i}}\;}{\min}\frac{1}{2}{\left|\left|w\right|\right|}^{2}+C\overset{m}{\underset{i=1}{\sum }}l_{\epsilon }\left(\xi_{i},\hat{\xi_{i}}\;\right). \end{array}$$

Introducing Lagrange multiplier *μ*_*i*_, we get

(4) $$\begin{array}{c} L\left(w,b,\alpha ,\hat{\alpha ,}\xi ,\hat{\xi },\mu ,\hat{\mu }\right)=\frac{1}{2}{\left|\left|w\right|\right|}^{2}+C\overset{m}{\underset{i=1}{\sum }}\left(\xi_{i},\hat{\xi_{i}}\right)-\overset{m}{\underset{i=1}{\sum }}\xi_{i}\mu_{i}-\overset{m}{\underset{i=1}{\sum }}\hat{\xi_{i}}\hat{\mu_{i}}+\overset{m}{\underset{i=1}{\sum }}\alpha_{i}\left(f\left(x_{i}\right)-y_{i}-\in -\xi_{i}\right)+\overset{m}{\underset{i=1}{\sum }}\hat{\alpha_{i}}\left(y_{i}-f\left(x_{i}\right)-\in -\hat{\xi_{i}}\right). \end{array}$$

Then, we make the partial derivative of $L\left(w,b,\alpha ,\hat{\alpha ,}\xi ,\hat{\xi },\mu ,\hat{\mu }\right)$ zero.

(5) $$\begin{array}{c} w=\overset{m}{\underset{i=1}{\sum }}\left(\alpha_{i}-\hat{\alpha_{i}}\right)x_{i}. \end{array}$$

Therefore,

(6) $$\begin{array}{c} f\left(x\right)=\overset{m}{\underset{i=1}{\sum }}\left(\alpha_{i}-\hat{\alpha_{i}}\right)k\left(x_{i},x\right)+b, \end{array}$$

where *k*(*x*_*i*_, *x*)  is a positive definite kernel function, *α*=(*α*_1_, *α*_2_,…,*α*_*m*_)^*T*^, and $\hat{\alpha }={\left(\hat{\alpha_{1}},\hat{\alpha_{2}},\ldots ,\hat{\alpha_{m}}\right)}^{T}$ and *b* are the parameters of the model. To achieve $\alpha_{i},\hat{\alpha_{i}}$, *i* = 1…*m*, the following objective function needs to be minimized:

(7) $$\begin{array}{c} \underset{i,j=1}{\sum }\left(\alpha_{i}-\hat{\alpha_{i}}\right)\left(\alpha_{j}-\hat{\alpha_{j}}\right)k\left(x_{i},x_{j}\right)+\varepsilon \overset{m}{\underset{i=1}{\sum }}\left(\alpha_{i}+\hat{\alpha_{i}}\right)-d\overset{m}{\underset{i=1}{\sum }}\left(\alpha_{i}-\hat{\alpha_{i}}\right), \end{array}$$

which is subject to the following expression:

(8) $$\begin{array}{c} \overset{m}{\underset{i=1}{\sum }}\left(\alpha_{i}-\hat{\alpha_{i}}\right)=0\text{ and }\alpha_{i},\hat{\alpha_{i}}\in \left[0,C\right], \end{array}$$

where *ε* and C are the hyperparameters.

The kernel *k*(*x*_*i*_, *x*) might have diverse forms. The most applied one is the Gaussian function *k*(*x*_*i*_, *x*)=exp(−||*x*_*i*_ − *x*||^2^/*σ*^2^), where *σ* > 0 is kernel's width. The training of SVR needs to solve the quadratic optimization problems given in equation (7) and (8) with two parameters. With the notion of support vector, the regression function given in equation (6) of SVR can be simplified as

(9) $$\begin{array}{c} f\left(x\right)=\overset{m}{\underset{x_{i}\in SV}{\sum }}\left(\alpha_{i}-\hat{\alpha_{i}}\right)k\left(x_{i},x\right)+b. \end{array}$$

### 2.3. Data Feature Extraction and Workflow

According to the running state data of robots, the content of each element in lubricating oil was collected and tested. Sampling principle considers robot model, running time, robot shaft position, and robot working condition.

- Each robot has six shafts, each shaft has lubricating oil, and the amount of lubricating oil varies with the position of the robot shaft. All the data of six axes are collected.
- The robot runs for 5,000 to 20,000 hours and is divided into distributed sampling groups.
- Group by robot load (10 to 300 kg).
- Select the robot candidates in the high-risk fault area (artificial judgment).

**Figure 3:**
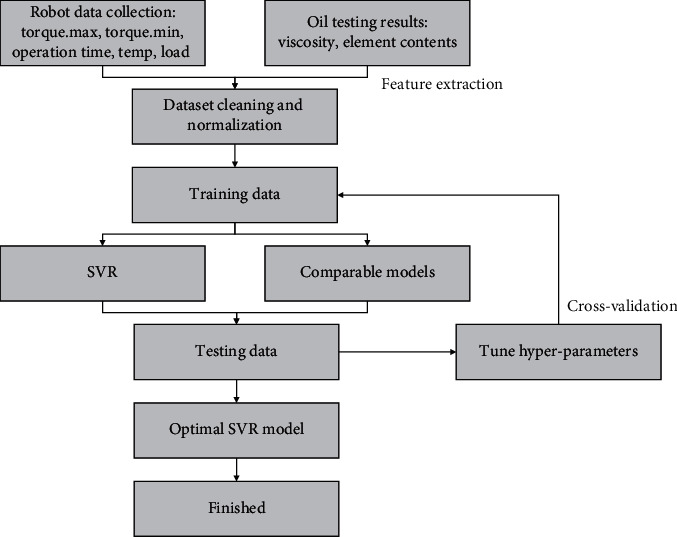
Overall workflow of the study.

Robot feature extraction of other running parameters:

- Torque extraction of each axis: the motion of the robot axis includes positive and negative rotations, and the negative maximum, positive maximum, and average value of torques in the stable operation period of the robot are extracted.
- Extract the maximum temperature and average temperature of each axis when the robot runs stably. Collect the lubricating oil samples and send them to the third party for testing, who would feed back the results, including the contents of iron, copper, aluminum, silicon, molybdenum, nickel, lead, chromium, etc. Lubricating oil itself contains silicon, molybdenum, nickel, lead, and chromium, excluding iron. According to the analysis of actual operation, the wear of the robot during operation results in the generation of iron in the lubricating oil. Therefore, the content of iron is the most obvious to characterize the lubricating oil state. The overall workflow is shown in Figure 3.

## 3. Results and Discussion

The running time, positive and negative torque, average torque, average temperature, and load are taken as a set of feature data, and the corresponding iron content is taken as the target data. All the numerical data are analyzed with quartiles. Any outliers higher than *Q*3 + 1.5*∗*IQR or lower than *Q*1 − 1.5*∗*IQR are removed separately. Thereafter, the data are normalized according to their dimensions using the formula (*X* − Min)/(Max − Min).

The whole dataset is randomly split into 80% and 20% as the training and testing datasets. The models are trained based on the SVR and the other comparable algorithms. The regression type is *ε*-SVR with the specific kernel function of Gaussian radial basis functions (RBF), since it is suitable for the nonlinear problem [20]. Six groups of cross-validation patterns are used, and the penalty coefficient C and width coefficient *γ* are optimized by the grid search method. As a result, the model performances are compared for the statistical interpretation and superior model selection [21]. The results are summarized in Table 1.

According to the table, four models are applied for forecasting. The root mean square error (RMSE) and the coefficient of determination (R-square) of linear regression and ridge regression are limited by their inherent complexities. The decision tree regressor is tested as the random forest, which has low RMSE and high R-square on the train dataset. However, the results on test dataset are not ideal. Obviously, it is hard to avoid overfitting even though the max_depth was limited less than two. Consequently, the SVR would be selected as the superior candidate with the optimized parameters. It exhibits balanced scores on both of the datasets.

**Figure 4:**
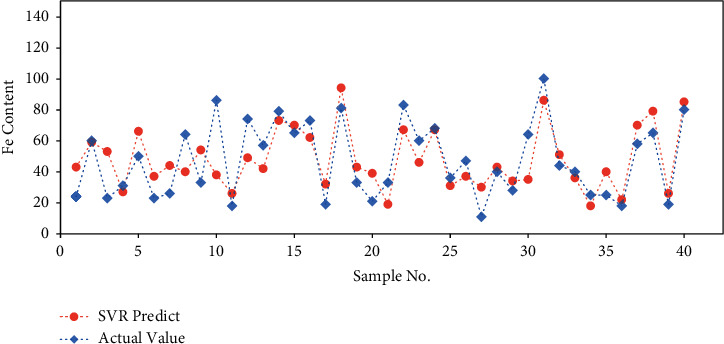
SVR model performance on train dataset.

**Figure 5:**
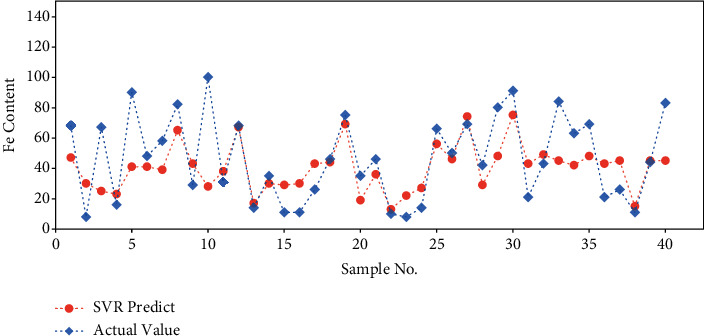
SVR model performance on test dataset.

The SVR prediction is further compared with the actual values on the train and test datasets separately (Figures 4 and 5). The output values are applied inverse normalization so that they can be exhibited with the original unit. Similarly, the model performs better on the train dataset than test dataset. The predicted results on test dataset are still close to the actual values with acceptable error.

## 4. Conclusions

Regarding the industrial robot lubricating oil replacement, the manufacturer recommends the replacement period to be 5 years. The average cost of changing robot lubricants is 10,000 CNY. Under the scale effect, according to the calculation of 3000 robots in BBAC, the replacement cost may reach to 30 million CNY. Meanwhile, the large number of replacement will take up the work force, resulting in difficulties to both equipment repairing and maintenance strategies. Regarding the periodic replacement, there would be serious excessive or insufficient maintenance, which would spend huge number of spare parts and work force cost. Herein, the present model can quantify the lubricating oil status and prepare the maintenance plan according to its actual lifetime. The optimized SVR would forecast the iron content with the relatively low normalized RMSE (0.15694) and high R-square (0.33637) that are better than other benchmark models. It may save 2 million CNY per year, and relieve the workload of front-line maintenance labors. This research is expected to bring some insight to the intelligent industry and predictive maintenance. Furthermore, the prediction model has the potential to be further tuned, which may be achieved by finely tailoring and supplementing with abundant data source in the future.

## Figures and Tables

**Table 1:** Summary of comparable model results.

Algorithm	Train RMSE	Test RMSE	Train R-square	Test R-square
Linear regression	0.16288	0.16744	0.32777	0.24465
Ridge regression	0.16413	0.16726	0.31736	0.24625
Random forest	0.14237	0.16295	0.48641	0.28457
SVR	0.14644	0.15694	0.45659	0.33637

## Data Availability

The data used to support the findings of this study are included within the supplementary information file.

## Supplementary Materials

Supplementary Materials The supplementary material contains the raw data (“Robot Lubricant Oil Data.txt”) utilized in the present study.
